# Influenza pH1N1 Virus Accumulated H275Y Mutation in Neuraminidase during Propagation in MDCK Cells

**DOI:** 10.1128/genomeA.01349-14

**Published:** 2014-12-24

**Authors:** Polina Mishel, Dmitrii Bychkov, Hannimari Kallio-Kokko, Miia Valkonen, Anu Kantele, Pirkko Mattila, Henrikki Almusa, Petri Jalovaara, Denis Kainov

**Affiliations:** a The Institute for Molecular Medicine Finland (FIMM), Helsinki, Finland; b Helsinki University Hospital Laboratory, HUSLAB, Helsinki, Finland; c Helsinki University Central Hospital, HUCS, Helsinki, Finland

## Abstract

Here, we sequenced the genome of the influenza A/Finland/741 M/2014(H1N1) virus and found that the virus accumulated oseltamivir resistance mutation H275Y in its neuraminidase during propagation in cell culture. This indicates that propagation in cell culture modifies virus genomes. The instability of influenza genomes should be taken into consideration during drug-sensitivity studies.

## GENOME ANNOUNCEMENT

Influenza A viruses evolve rapidly and cause global pandemics and annual epidemics, which represent a serious social and economic problem. Influenza infection causes mild and severe diseases. Vaccinations and antiviral therapeutics, such as oseltamivir, remain the principal method of preventing severe flu and its complications. The viruses are diagnosed in public health laboratories using molecular assays which are based on nucleic acid amplification, serology, antigen detection, and cell culture assays.

The recent influenza pandemic was caused by the pH1N1 virus. The genome of the pH1N1 virus consists of 8 single-stranded RNA segments and encodes 12 proteins: hemagglutinin (HA), neuraminidase (NA), nucleocapsid protein (NP), M proteins (M1 and M2), nonstructural proteins (NS1), nuclear export protein (NEP), polymerase subunits (PA, PB1, PB2), and auxiliary proteins PA-X and PB1-F2. We have shown recently that pH1N1 viruses accumulate mutations in virus genomes during propagation in cell culture (1). In particular, viruses accumulate E119K and D151E/N mutations in NA which are associated with oseltamivir-resistance.

Here, we demonstrate that the A/Finland/741 M/2014 virus accumulated H275Y mutation in HA, which is also known to be associated with oseltamivir-resistance (2). Briefly, we collected nasopharyngeal aspirate from a male patient with a mild infection. The patient did not receive an oseltamivir treatment before sampling. Due to the low concentration of viral RNA in the sample, we amplified the virus in MDCK cells. We sequenced the A/Finland/741 M/2014 virus using the procedure described in by Lakspere et al. (3). We found H275Y mutation in NA. We then sequenced the NA fragment of the original virus with 5′-TAAAGTACAACGGCATAATAA and 5′-AGTTATCCCTGCACACACATG primers and searched for H275Y mutation. However, the original virus had histidine at position 275. We concluded that the virus accumulated drug-resistant mutation during propagation in cell culture.

This and other mutations, such as mutations in HA (N/D222G, HA1 numbering), NA (D151E/N, S95N, N386K, K397N, G398E, N449K), NS1 (D2N, I18V, E55K), and PB1 (V113I) should be taken into consideration when pH1N1 viruses are amplified in cell culture because they could interfere with the interpretation of drug-sensitivity and virulence surveillance studies**.**

### Nucleotide sequence accession numbers.

The full genome sequence of influenza A/Finland/741 M/2014(H1N1) virus has been deposited in GenBank under accession numbers KM366687 to KM366694.
